# Investigation of the total anticholinergic load of reported anticholinergic drug-related adverse events using the Japanese adverse drug event report database: a retrospective pharmacovigilance study

**DOI:** 10.1186/s40780-025-00413-w

**Published:** 2025-01-31

**Authors:** Yusuke Kan, Maki Doi, Yoshihiro Uesawa

**Affiliations:** 1 Nanohana Pharmacy, Nanohana Hokkaido, Inc, Tomakomai, 053-0021 Japan; 2 Department of Academics, Medical System Network, Inc, Sapporo, 060-0010 Japan; 3 Department of Medical Molecular Informatics, Meiji Pharmaceutical University, Kiyose, 204-8588 Japan

**Keywords:** JADER, Japan anticholinergic risk scale, Total anticholinergic load, Anticholinergic syndrome

## Abstract

**Background:**

The Anticholinergic Risk Scale and Total Anticholinergic Load were developed to assess the risks associated with anticholinergic drugs. Recently, the Japan Anticholinergic Risk Scale was introduced; however, the total anticholinergic load for adverse events has not been clarified, and the criteria for risk assessment in clinical practice have not been established. In this study, we used data from the Japanese Adverse Drug Event Report (JADER) database provided by the Pharmaceuticals and Medical Devices Agency to determine the total anticholinergic load associated with reported adverse events related to anticholinergic syndrome.

**Methods:**

Using JADER data from April 2004 to September 2023, we investigated the association between drugs included in the J-ARS and adverse events related to anticholinergic syndrome. In addition, we calculated the total anticholinergic load for each case involving a drug recorded in the JADER database and compared it with other adverse events associated with anticholinergic effects.

**Results:**

Based on the JADER data, we observed an association between anticholinergic syndrome-related adverse events and the drugs listed in the J-ARS, confirming the feasibility of calculating the total anticholinergic drug burden for each case. In the group reporting anticholinergic syndrome–related adverse events, the mean ± standard deviation of the total anticholinergic load was 4.20 ± 3.09.

**Conclusions:**

The mean total anticholinergic load of anticholinergic syndrome-related adverse events obtained from the JADER database in this study supports the development of a comprehensive risk assessment of anticholinergic drugs in clinical practice.

**Supplementary Information:**

The online version contains supplementary material available at 10.1186/s40780-025-00413-w.

## Background

Anticholinergic drugs, including antihistamines and antipsychotics, are widely used clinically and are known to cause various adverse events [1]. Approximately half of elderly individuals are reported to take at least one anticholinergic drug [2], and adverse events in elderly patients at home are relatively common [3]. Therefore, it is important to manage the effects of anticholinergics in the home and outpatient settings.

The anticholinergic risk scale (ARS) [4] and total anticholinergic load (TAL) [5, 6, 7] were developed to assess the effects of anticholinergic drugs. Although individual drug risk assessment is important for anticholinergic risk assessment, in clinical practice, many patients take multiple medications, and the use of multiple anticholinergic agents increases patient risk. Therefore, determining the TAL may be beneficial because it enables a comprehensive risk assessment for each patient, and switching to a lower-scoring medication is recommended if a higher-scoring medication is used.

The TAL can be used to assess a patient’s overall risk and determine the risk of anticholinergic effects of the overall pharmacotherapy. A higher TAL has been associated with poorer physical function and cognitive decline in the elderly [8]. However, to date, issues have been identified, including the low level of agreement observed among the various ARS developed to date [9, 10].

In May 2024, the Japanese Society of Geriatric Pharmacy developed the Japan Anticholinergic Risk Scale (J-ARS) [11] It is recommended that the J-ARS be used in clinical practice to assess the anticholinergic risk in individual patients. However, the TAL calculated from the J-ARS does not have established criteria to serve as an indicator for comprehensive risk assessment, resulting in a lack of evaluation of the overall risk of anticholinergic effects in clinical pharmacotherapy.

We hypothesized that it is possible to calculate TALs based on the J-ARS for adverse event cases in Japan using the Japanese Adverse Drug Event Report (JADER) database provided by the Pharmaceuticals and Medical Devices Agency. To confirm whether the JADER database is applicable to the analysis of anticholinergic syndrome-related adverse events and anticholinergic drugs, we examined its relevance. We also analyzed the TAL in cases of J-ARS-related drug use. This study aimed to determine the TAL in cases of anticholinergic drug-related adverse events using the JADER for drugs evaluated in the J-ARS.

## Methods

### Japan anticholinergic risk scale

Among the drugs available in Japan (both prescription and over-the-counter), only oral and transdermal drugs for systemic action are included in the J-ARS, with scores assigned to 158 drugs [11]. The scores for each drug were based on pharmacodynamic evaluations and expert opinion. Of these drugs, 37, 27, and 94 were rated with scores of 3, 2, and 1, respectively. In general, the ARS is primarily applied to the elderly; however, because younger people may also be at an increased risk of adverse drug reactions depending on their underlying medical conditions, there is no age distinction in the J-ARS assessment.

### Database and data table construction

We used the JADER database, a large Japanese database, for voluntary adverse event reporting. This publicly available database can be downloaded free of charge after confirming the terms of use [12]. The downloaded database contained data from April 2004 to September 2023. The database was anonymized, personal information was removed when recorded, and an identification (ID) number was assigned to each case to identify each individual. Therefore, database users cannot identify the individuals recorded in the database.

**Fig. 1:**
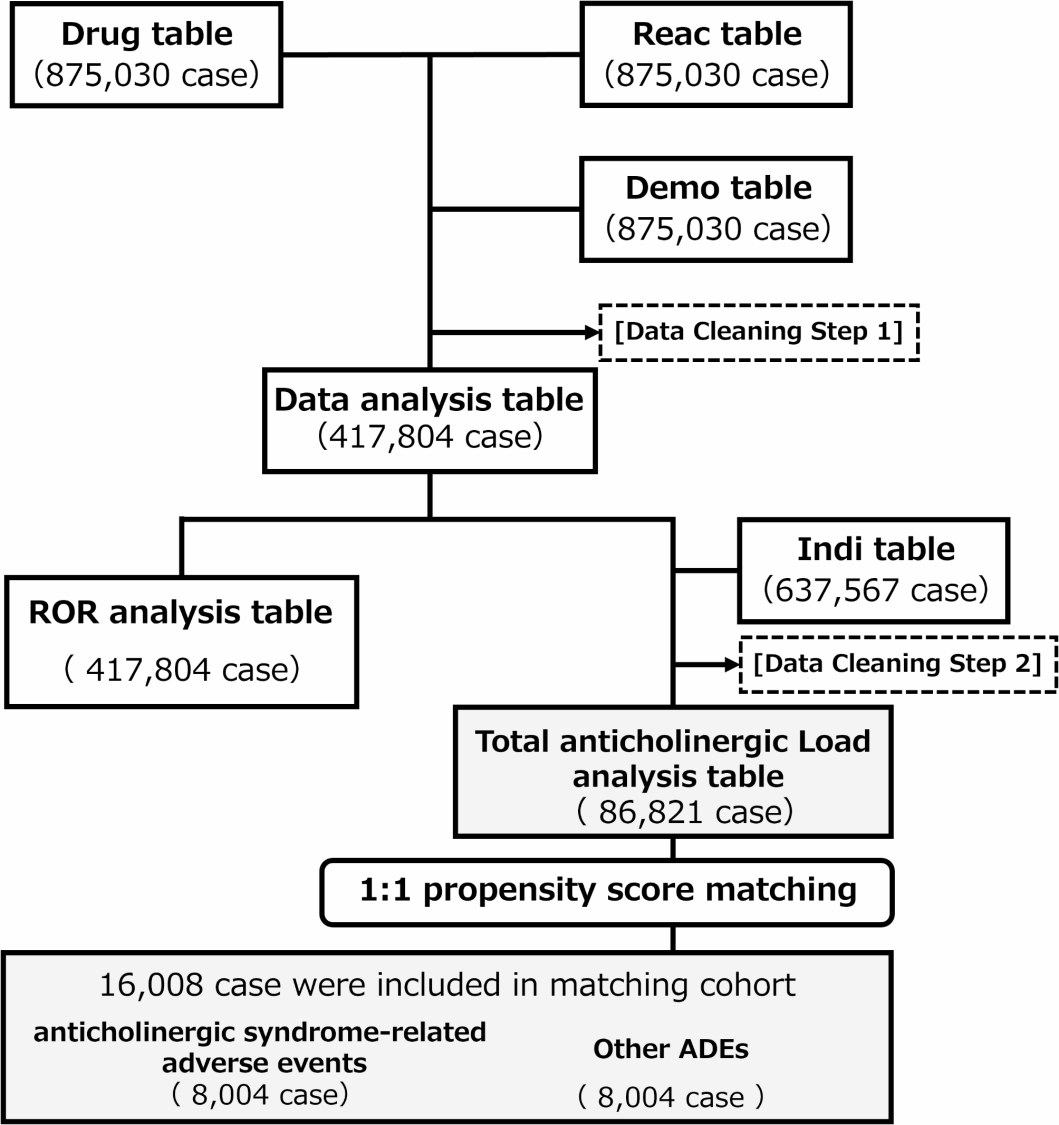
Flowchart for creating data tables for analysis. [Data Cleaning Step 1]: Excluded cases where drugs were reported as medications other than the drug under investigation, where drugs were administered via routes other than oral or dermal, and cases with unknown age or gender. [Data Cleaning Step 2]: Excluded cases where the drug under investigation was not reported as a target drug in the J-ARS

The data necessary for the analysis were extracted and an analysis table was created (Fig. 1). Cases with unknown gender and age were excluded [13]. Although JADER includes the names of active pharmaceutical ingredients, a single ingredient may be formulated in multiple dosage forms. To prevent assigning scores to dosage forms not originally covered by J-ARS, we adhered to the J-ARS exclusion criteria. Medications with routes of administration other than oral and transdermal were also excluded, which was consistent with the J-ARS criteria. In the JADER database, the terms “suspect drug,” “concomitant drug,” and “interaction” were used to record the involvement of the reported drug in the drug table. “Suspected drugs” are considered to have a potential causal relationship with adverse events, thus aiding in identifying cases related to specific adverse outcomes. However, focusing solely on “suspected drugs” may yield only partial scores for reported cases, complicating comprehensive case evaluations. Conversely, “concomitant medications” and “interactions” encompass all medications used in the reported cases. Including these in the TAL calculation allows for a more detailed evaluation on a case-by-case basis. In this study, all of these categories were used to calculate the TAL for each reported case, and only the “suspect drug” was used to determine the association between J-ARS drugs and adverse events related to anticholinergic effects.

### Adverse events related to anticholinergic syndrome

We used standard MedDRA search terms (SMQ) and category terms from MedDRA/J ver26.0 [14]. Categories were assigned to the preferred terms (PT) included in “anticholinergic syndrome” (SMQ: 20000048). Category A refers to cases with “anticholinergic syndrome” recorded as yhe PT. Category B refers to PTs related to the nervous system, such as dizziness and somnolence. Category C refers to PTs related to the mental system, such as delirium and hallucinations. Category D refers to PTs related to other anticholinergic syndromes, such as dry mouth and urinary retention. In this study, “anticholinergic syndrome” was defined as a case with a documented PT listed in category A or with at least one PT each listed in categories B, C, and D. Cases with each of the other categories recorded were designated “anticholinergic syndrome-related adverse events.”

### Drugs subject to the Japan Anticholinergic Drug Risk Scale and characteristics of adverse events in the JADER database

To observe the association between J-ARS drugs and adverse events, we used a signal detection method commonly used to indicate the association between drugs and adverse events [15]. The reported odds ratio (ROR) and P-value from Fisher’s exact test were calculated from a 2 × 2 contingency table. To stabilize the parameter estimates, all cells were adjusted by adding 0.5 (Haldane–Anscombe 1/2 correction) [16]. Adverse events in at least 100 reported cases were included in the observation [17]. A scatter plot (volcano plot) was created using the inverse of the ordinary logarithm of the calculated *P*-value from Fisher’s direct exact test (-log [*P*-value]) on the vertical axis and the natural logarithm of the ROR (ln ROR) on the horizontal axis [18].

### Confounder control and matching

To mitigate the effects of comorbidities, we integrated the Indication table—which records primary diseases—with the TAL analysis table. For propensity score matching, we adjusted for nine potential confounding factors, including demographic variables and comorbidities suspected to be associated with TAL (Supplementary Material Table S1). We estimated the propensity score using logistic regression analysis and then performed 1:1 nearest-neighbor matching. We assessed the balance between the groups with anticholinergic syndrome–related adverse events and other adverse events, before and after propensity score matching, by examining standardized differences. A standardized difference of less than 10% was deemed indicative of a well-balanced distribution of confounding factors between the two groups [19].

### Calculation of the total anticholinergic load per case

Among the cases recorded in the JADER database, we extracted those that contained J-ARS drugs. It has been reported that a higher total ARS score correlates with an increased incidence of anticholinergic side effects [4]. Therefore, to clarify the TAL reported for anticholinergic syndrome–related adverse events and to use it as a benchmark for preventing serious adverse events, we aggregated the J-ARS scores from medications in the extracted cases and calculated each case’s TAL [12]. The mean ± standard deviation of the TAL was calculated for cases reporting anticholinergic syndrome-related adverse events and cases reporting other adverse events (ADEs). The Steel test was performed to determine the difference in the mean values of TALs calculated for each recorded category group, with cases reporting events other than adverse events related to anticholinergic syndrome being considered the control group.

### Statistical analysis

All data analyses were performed using JMP Pro 17.2.0 software (SAS Institute Inc., USA). P-values < 0.05 were considered statistically significant.

## Results

### Construction of data tables for analysis

In the JADER database used in this study, 875,030 cases were recorded for drug information, adverse reaction information, and case list tables. These tables were combined to create data tables for the analysis. Analysis dataset with 417,804 cases (Fig. 1). Data with unclear age and sex were excluded. Data from routes of administration other than oral and dermal routes were also excluded to align with the routes covered by the J-ARS.

### Japan anticholinergic drug risk scale and characteristics of adverse events

**Fig. 2:**
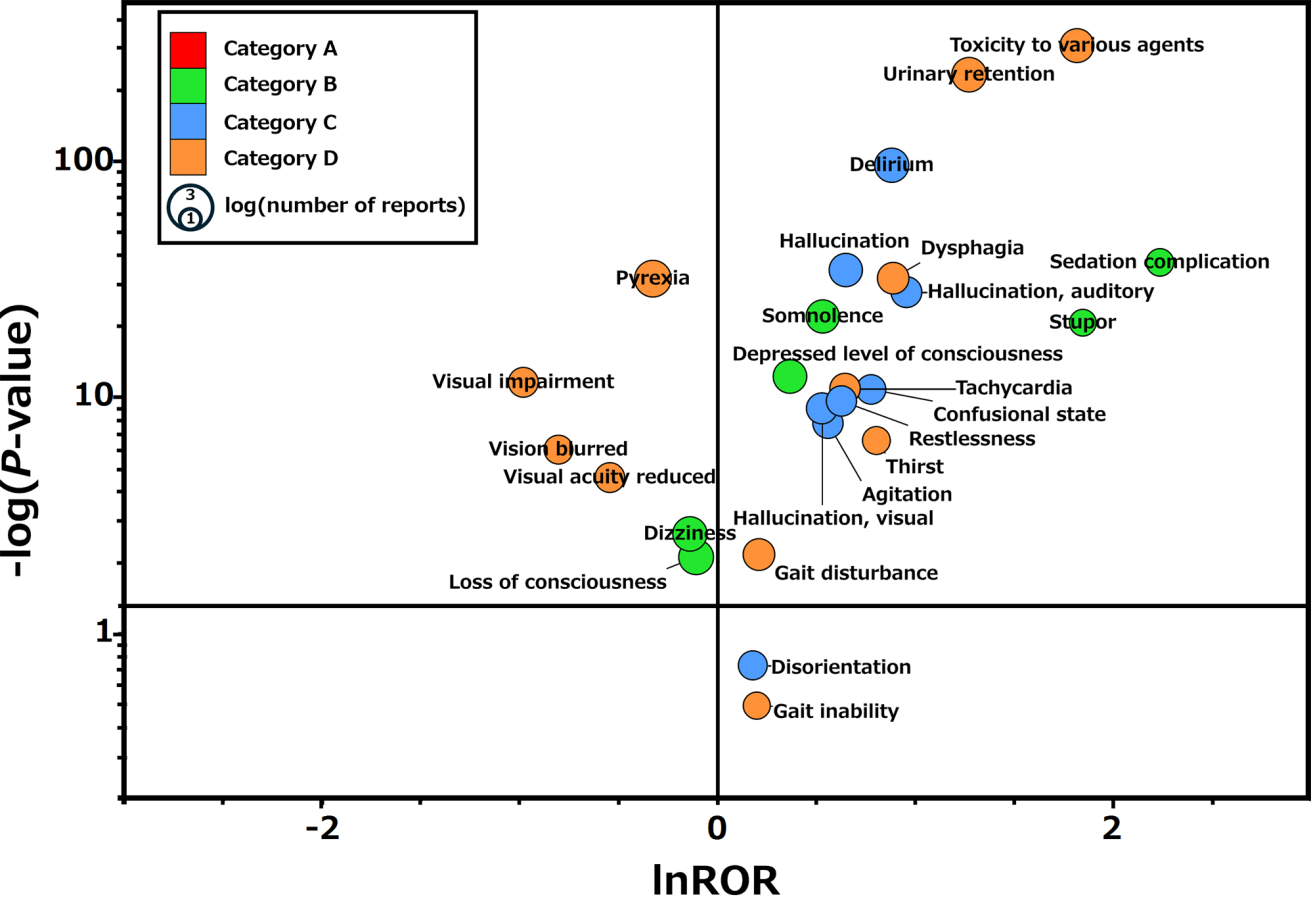
Volcano plot showing the association between J-ARS drugs and anticholinergic syndrome-related adverse events in the JADER database. The horizontal axis represents the natural logarithm of the reported odds ratio (lnROR), and the vertical axis shows the reciprocal of -log(P-value) obtained from Fisher’s exact test. The horizontal line indicates the criterion of -log(P-value) = 1.3 (*P* = 0.05). Color-coded categories represent different types of adverse events related to anticholinergic syndrome

We hypothesized that the JADER database could be used to calculate TALs based on the J-ARS for adverse event cases, consistent with drug use patterns in Japan. To confirm whether the JADER database is applicable to the analysis of anticholinergic syndrome-related adverse events and anticholinergic drugs, we created a volcano plot showing the association between J-ARS drugs and anticholinergic syndrome-related adverse events (Fig. 2).

The ROR analysis table includes 112,696 cases in which J-ARS drugs were reported as “suspect drugs.”

In the volcano plot, the right side of the horizontal axis indicates the AEs that are more frequently associated with J-ARS drugs. In contrast, the upper part of the vertical axis indicates statistically significant adverse events.

In the data recorded in the JADER database, associations with J-ARS drugs were observed in the majority of neurological, psychiatric, and other anticholinergic syndrome-related adverse events (Supplementary Material Table S2). However, other anticholinergic syndrome-related adverse events, such as those related to vision, were not associated with JARS-targeted drugs. The results indicate that the JADER data used in this study are consistent with the association between J-ARS drugs and anticholinergic syndrome-related adverse events, as adverse events specific to anticholinergic effects are generally associated with J-ARS drugs. Therefore, the data recorded in the JADER database could be used to calculate the TAL for adverse events associated with anticholinergic syndromes caused by J-ARS drugs.

### Matching of the analysis population

A total of 86,821 cases were extracted for the case-specific TAL calculation table. Of these, 8,004 cases in the anticholinergic syndrome–related adverse event group were matched 1:1 with 8,004 cases in the other adverse event group using propensity score matching. After this matching, the standardized difference was less than 10%, indicating that an adequate balance had been achieved between the groups (Supplementary Material Table S3).

### Calculation of the TAL per case

In the total anticholinergic drug burden analysis table, among the cases that identified J-ARS–targeted drugs as “suspected drugs,” 8,004 reported anticholinergic syndrome–related adverse events after matching. Of these, the most frequently reported single category was Category D, accounting for 24.23%, followed by single Category B at 11.55% and single Category C at 11.45%. Meanwhile, 0.01% of the cases reported anticholinergic syndrome (Category A or Category B, C, and D) (Table 1).

Using the total anticholinergic drug burden analysis table, we calculated the TAL for adverse events related to anticholinergic syndrome caused by the J-ARS target drugs (Supplementary Material Table S4). For the group of cases reporting cholinergic syndrome (Category A or Categories B, C, and D), the mean ± standard error of TAL was 4.20 ± 3.09. When the group reporting other adverse events was used as the control, there was no significant difference in the mean TAL for the group that developed anticholinergic syndrome. Conversely, in the group of cases reporting anticholinergic syndrome–related adverse events (Categories B, C, or D alone; or combinations of Categories B and C, B and D, or C and D), each subgroup exhibited a significantly higher TAL than that exhibited by the group reporting other adverse events (Table 2).

**Table 1:** Percentage of reported anticholinergic syndrome-related adverse events

	case	percentage
Anticholinergic Syndrome		
Category A	1	0.01%
Category B&C&D	19	0.12%
Anticholinergic Syndrome-Related ADEs		
Category B	1849	11.55%
Category C	1833	11.45%
Category D	3879	24.23%
Category B&C	118	0.74%
Category B&D	207	1.29%
Category C&D	98	0.61%
Other ADEs		
	8004	50.0%

In addition, higher mean TAL values were observed in the case groups that reported more than one category (B and C, B and D, or C and D) than in those that reported only one category (B, C, or D). The number of concomitant J-ARS drugs in each case group tended to be higher in case groups that reported multiple categories. When the same analyses were performed by route of administration, the oral route exhibited a trend similar to that observed in the that did not consider administration routes. Conversely, the transdermal route showed significant differences in certain categories (Supplementary Material Table S5).

**Table 2:** TAL and number of concomitant medications in reported cases of anticholinergic syndrome-related adverse events

Type	Category	Total anticholinergic load			J-ARS drugs
		Mean	SD	*P*-value*	Average combination drugs
Anticholinergic syndrome	Category A or Category B&C&D	4.2	3.09	0.0777	2.80
Single Category	Category B	2.88	2.29	< 0.0001	2.15
	Category C	3.28	2.62	< 0.0001	
	Category D	3.16	2.6	< 0.0001	
Multiple Category	Category B&C	3.36	2.28	< 0.0001	2.40
	Category B&D	3.61	2.79	< 0.0001	
	Category C&D	3.71	2.41	< 0.0001	
	others ADEs	2.69	2.34	-	2.03

*Steel’s multiple comparison test was performed vs. other ADEs

## Discussion

This study retrospectively investigated the TAL of J-ARS in adverse events associated with anticholinergic syndromes using the JADER database. To our knowledge, this is the first report of TALs and adverse events based on the J-ARS.

Using a volcano plot, we observed an association between JADER-recorded J-ARS medications and anticholinergic syndrome-related adverse events. An association was demonstrated between J-ARS drugs and adverse events related to anticholinergic syndrome, as recorded in the JADER database. The use of spontaneously reported adverse event databases has been shown to facilitate the analysis of relationships between anticholinergic drugs and adverse events [20]. The results of this study suggest that selecting J-ARS drugs recorded in the JADER database may also enable the evaluation of these relationships effectively. However, because the JADER data is often reported by healthcare professionals, objective adverse events are more likely to be reported, so subjective adverse events are less likely to be reported [21]. Some subjective adverse events, such as visual impairment, exhibited similar trends. Spontaneously reported adverse event databases are subject to reporting bias because not all adverse events that occur in patients are reported. Therefore, it is possible that this analysis failed to establish a relationship between certain adverse events and J-ARS drugs.

Using the J-ARS drugs recorded in the JADER database, we calculated the approximate TAL at the time of adverse event occurrence. Previous studies have shown that among older adults aged 65 years and older, an anticholinergic cognitive burden (ACB) score of ≥ 4—which assesses central anticholinergic effects—is associated with cognitive decline and poor clinical outcomes [22]. In the present study, by analyzing a population that included young individuals, we established TAL reference values applicable to a broad range of patients. Although the ACB is suitable for patients at risk of cognitive impairment, it may not be ideal for a wide patient population. The scoring of ARS and ACB does not necessarily align, necessitating caution in direct comparisons of these cumulative scores. Nevertheless, in terms of the onset of anticholinergic syndrome–related adverse events—a previously unclear area—TAL exhibited a trend similar to that of cumulative ACB. Anticholinergic drug-related adverse events commonly encountered in clinical practice are not limited to cognitive function; a wide range of adverse events have been experienced [23]. A wider range of information on AEs is therefore necessary, particularly when performing comprehensive risk assessments of anticholinergic agents in clinical settings. In this study, TALs were calculated for each category of adverse events associated with the broad anticholinergic syndrome. The average calculated TALs may provide an indicator for the overall risk assessment of anticholinergic syndrome-related adverse events in J-ARS drugs.

The concomitant use of multiple anticholinergic medications can lead to overlapping anticholinergic effects on both the central and peripheral nervous systems, accentuating the cumulative effect [24]. Our results not only show a trend toward higher TAL values in cases reporting multiple categories compared to those reporting a single category, but also a trend toward a higher number of concomitant medications in the J-ARS target drugs. Consistent with previous reports, we observed that patients with higher TALs tended to develop various anticholinergic symptoms owing to the cumulative effect. This suggests that the risk assessment of anticholinergic drugs should not only include the selection of lower-risk anticholinergic drugs but also the selection of concomitant medications that lower the TAL. Since the J-ARS includes not only prescription drugs, but also the active ingredients of over-the-counter drugs, the results of this study may be useful for a comprehensive risk assessment of anticholinergic drugs in prescription and over-the-counter drugs. Therefore, the results of this study may be useful for the comprehensive risk assessment of anticholinergics in prescription and over-the-counter drugs.

This study has several limitations. First, it is based on spontaneous reporting and is subject to over-reporting, under-reporting, missing data, lack of cases with unreported adverse events, and the presence of confounding factors [25, 26]. Additionally, some adverse events were less likely to be reported. Therefore, this study established a process to determine whether data from the JADER database could be used to calculate the TAL by understanding an association between J-ARS drugs and adverse events. Moreover, the results of this study are based only on J-ARS drugs, so risk assessment cannot be performed for anticholinergic drugs not covered by the J-ARS. Because the J-ARS assigns scores directly to medications, it does not fully consider factors such as pharmacokinetics. Therefore, it is pertinent to carefully account for changes that may affect pharmacokinetics on an individual patient basis. In addition, the results of this study may not be applicable to adverse events associated with some anticholinergic syndromes, such as visual impairment, because the JADER trial used in this study did not show an association with J-ARS drugs.

## Conclusions

Using the reported cases of anticholinergic syndrome associated with J-ARS drugs listed in the JADER database, we calculated the mean TAL value. Our results may aid in the comprehensive risk assessment of anticholinergic drugs in terms of the TAL, for which no cut-off values have yet been established. Based on these results, further analysis of the TAL for J-ARS-targeted drugs using clinical data is warranted.

## Electronic supplementary material

Below is the link to the electronic supplementary material.

Supplementary Material 1

## Data Availability

All data generated or analyzed during this study are included in this published article.

## Abbreviations

ARS: anticholinergic risk scale
TAL: total anticholinergic load
J-ARS: Japan Anticholinergic Risk Scale
JADER: Japanese Adverse Drug Event Report database
SMQ: standard MedDRA search terms
PT: preferred terms
ROR: reported odds ratio
ADEs: adverse events
